# Author Correction: High frequency temperature variability reduces the risk of coral bleaching

**DOI:** 10.1038/s41467-018-04741-4

**Published:** 2018-06-05

**Authors:** Aryan Safaie, Nyssa J. Silbiger, Timothy R. McClanahan, Geno Pawlak, Daniel J. Barshis, James L. Hench, Justin S. Rogers, Gareth J. Williams, Kristen A. Davis

**Affiliations:** 1 Department of Civil and Environmental Engineering, University of California, Irvine, CA 92697 USA; 2 Department of Biology, California State University, Northridge, 18111 Nordhoff Street, Northridge, CA 91330-8303 USA; 3 Department of Ecology and Evolutionary Biology, University of California, Irvine, CA 92697 USA; 4 Marine Programs, Wildlife Conservation Society, 2300 Southern Boulevard, Bronx, NY 10460 USA; 5 Department of Mechanical and Aerospace Engineering, University of California San Diego, 9500 Gilman Drive, MC0411, La Jolla, CA 92093 USA; 6 Department of Biological Sciences, Old Dominion University, Mills Godwin Building 110, Norfolk, VA 23529 USA; 7 Nicholas School of the Environment, Duke University, 135 Duke Marine Lab Road, Beaufort, NC 28516 USA; 8 Department of Civil and Environmental Engineering, Stanford University, 473 Via Ortega, Y2E2 Rm 126, Stanford, CA 94305 USA; 9 School of Ocean Sciences, Bangor University, Anglesey, LL59 5AB UK

Correction to: *Nature Communications* 10.1038/s41467-018-04074-2, published online 26 April 2018

The original version of the Article was missing an acknowledgement of a funding source. The authors acknowledge that A. Safaie and K. Davis were supported by National Science Foundation Award No. 1436254 and G. Pawlak was supported by Award No. 1436522. This omission has now been corrected in the PDF and HTML versions of the Article.

