# Supplementary material for: High frequency temperature variability reduces the risk of coral bleaching
Source: Nat Commun. 2018 Apr 26;9:1671. doi: 10.1038/s41467-018-04074-2 (PMC5920114; doi:10.1038/s41467-018-04074-2)
Supplement: Supplementary file 1 — Supplementary Information [file 41467_2018_4074_MOESM1_ESM.pdf]

**High-frequency temperature variability reduces the risk of coral bleaching**

**Supplementary Information**

Safaie et al.

## Supplementary Note 1: High-frequency Spectral Content

Power Spectral Density (PSD) estimates of the 118 temperature time series reveal that all had significant diurnal and semidiurnal spectral peaks, emphasizing the ubiquity of significant high-frequency variance throughout our data set (Supplementary Fig. 1). The majority of time series also showed significant peaks corresponding to the shallow water terdiurnal and quarterdiurnal frequencies (99.2% and 92.4% of time series, respectively). Finally, 43 (30.5%) of the time series showed significant spectral peaks corresponding to long term, annual frequencies (1/90 – 1/540 cpd). Statistically significant spectral peaks are indicated in Supplementary Figure 1 to illustrate the persistence of high frequency water temperature variability among many different types of coral reef environments. Regardless of oceanographic region or latitudinal gradient, there is a considerable amount of temperature variability within diurnal and shorter periods that reefs are exposed to.

## Supplementary Note 2: Seasonal Variation in Daily Temperature Range

The results of Kruskal-Wallis tests for 113 of 118 (96%) temperature records (all except MD1, MD2 from Abu Madafi, Saudi Arabia, and P13, P23, and P32 from Palmyra Atoll, United States), allow us to conclude that daily temperature range (DTR) values are significantly different in distribution across the four astronomical seasons ($p < 0.01$), where seasons were defined such that each season spanned 3 complete months, and austral and boreal summers were December through February and June through August, respectively. This seasonality is illustrated for six representative time series as yearly composite averages in column **(b)** of Figure 2. The variable width of the pink patch in each yearly composite time series illustrates the variability of DTRs at these sites, while the red lines indicate the seasonally-averaged DTR. Maximum DTR values are most common during late spring to summer (boreal and austral) months (Supplementary Fig. 3), when solar heating of the ocean surface is largest, with the average summer DTR 1.2-2.1 times as large as the average winter DTR.

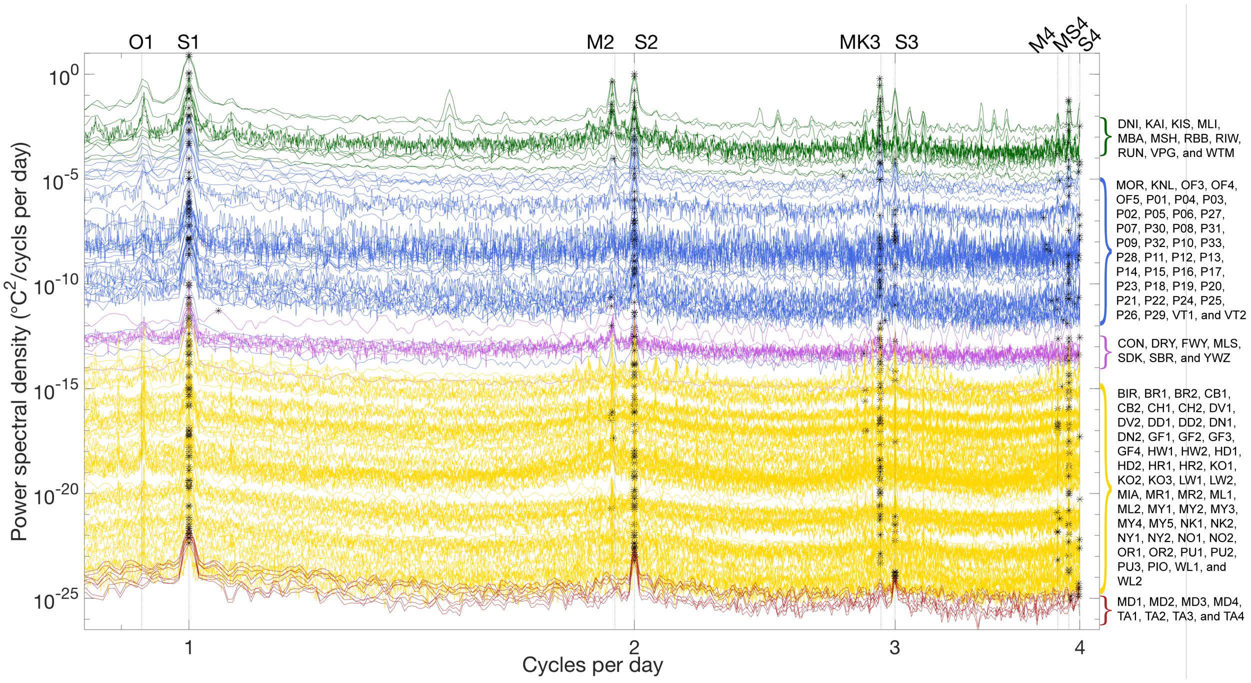


**Supplementary Figure 1| Power spectra at high-frequencies.** Power Spectral Density (PSD) estimates of water temperature from every time series, focused on the high frequency (0.727 to 4 cpd) part of the spectrum. PSD estimates are sequentially offset by $10^{-0.2}$ °$C^2$/cpd, and significant peaks are marked with an asterisk. The 3-letter abbreviations for each temperature time series are listed following their PSD estimates, and grouped by oceanographic region.

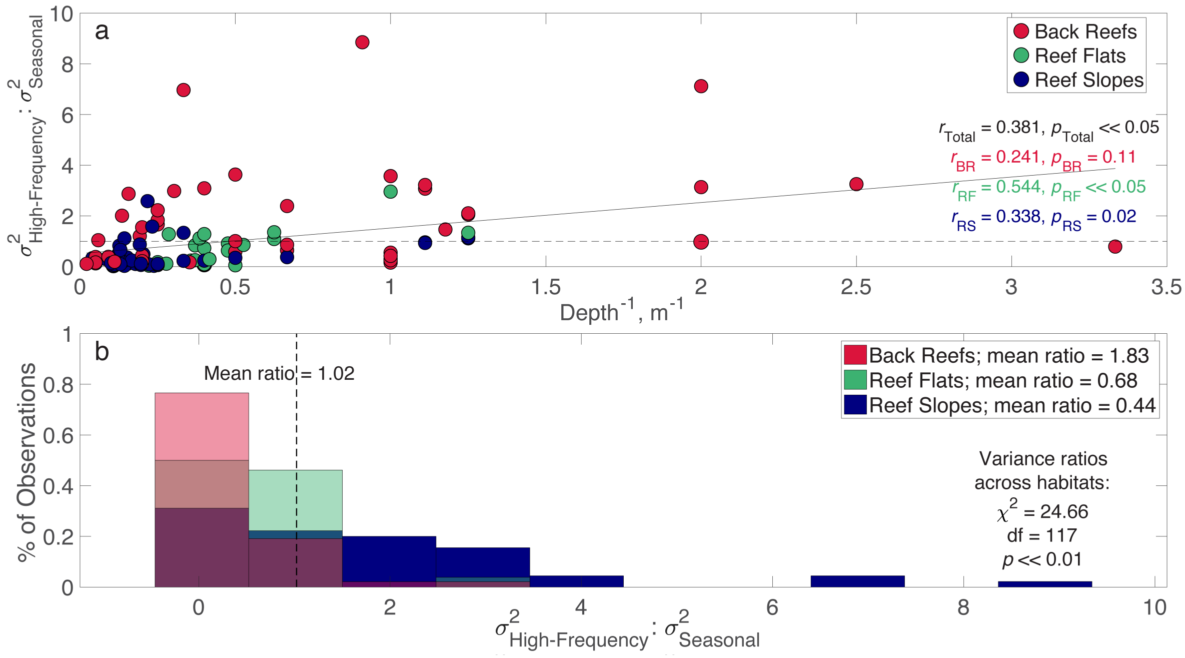


**Supplementary Figure 2| Ratios of high-frequency to seasonal variance by habitat. For all temperature time series,** high-frequency (1/33 to 1/6 h) and seasonal (1/7 to 1/84 d) variance were computed via integration of power spectral densities within these respective frequency bands. a) Variance ratios plotted against depth and colored by reef habitat, along with a linear regression line (solid black) and unity (dashed black); b) Histograms of the variance ratios are shown grouped by reef habitat, and the results of a Kruskal-Wallis test are indicated.

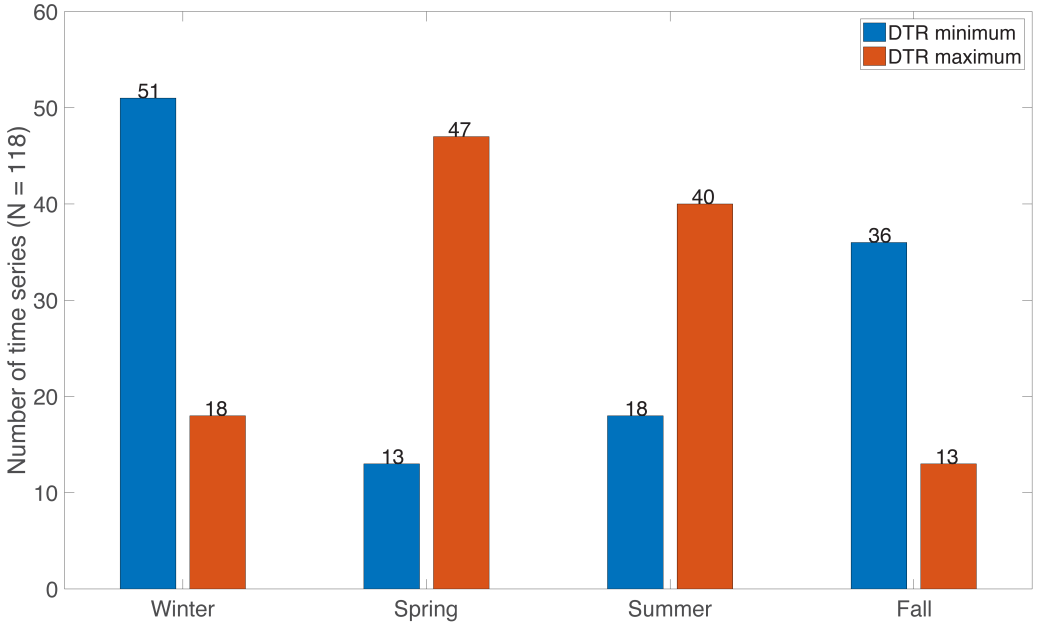


**Supplementary Figure 3| DTR extrema by season.** Orange bars: counts of time series with maximum DTR values in winter (18), spring (47), summer (40), and fall (13); blue bars: counts of time series with minimum DTR valuess in winter (51), spring (13), summer (18), and fall (36). Seasons were distinguished among northern and southern hemispheres, and arranged such that austral and boreal summers were December through February and June through August, respectively.

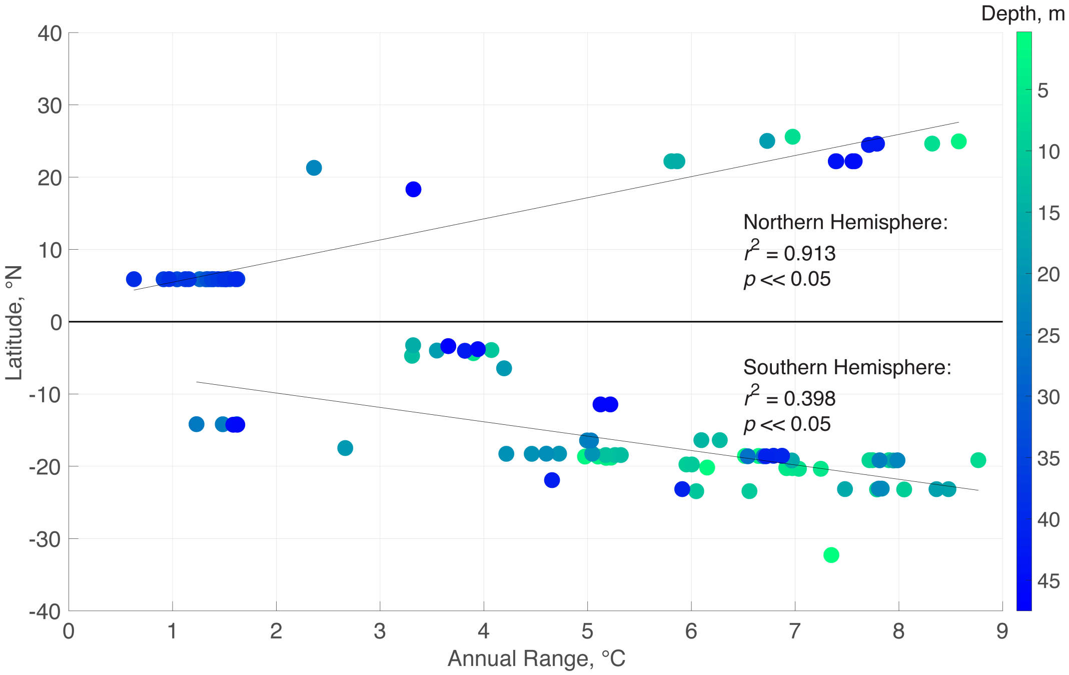


**Supplementary Figure 4| Latitude vs annual temperature ranges.** Latitudinal gradient in annual temperature ranges for every time series, arranged by hemisphere and colored according to *in situ* logger depth (m). Linear correlations and regression lines are shown for each hemisphere.

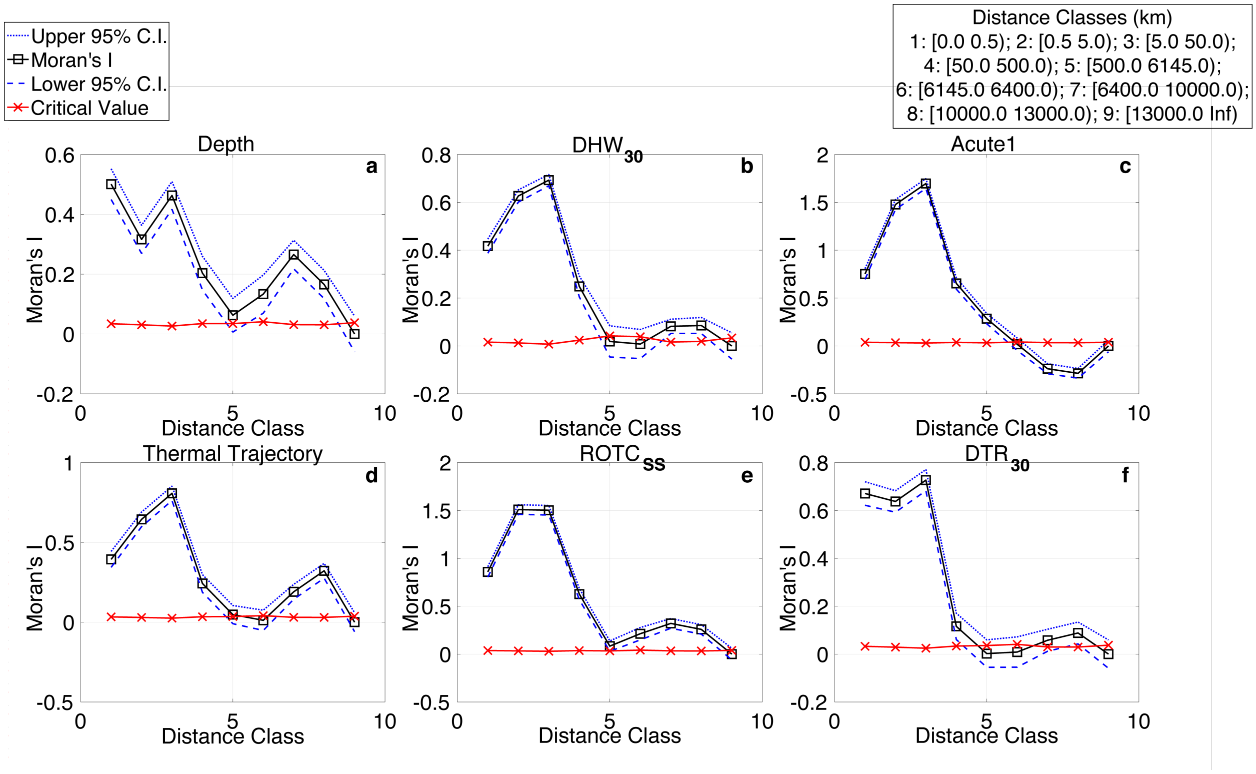


**Supplementary Figure 5| Moran's I Correlograms.** For the *in situ* covariates in the highest-ranked OLR model, Moran's I was computed using distance classes (km) of 1: [0, 0.5); 2: [0.5, 5.0); 3: [5.0, 50.0); 4: [50.0, 500.0); 5: [500.0, 6145.0); 6: [6145.0, 6400.0); 7: [6400.0, 10000.0); 8: [10000.0, 13000.0); 9: [13000.0, ∞). 95% confidence intervals are shown, and critical values for Moran's I were computed using a correction for small sample sizes. Covariates in the highest-ranked *in situ* model include **a)** Depth, **b)** $DHW_{30}$, **c)** Acute1, **d)** Thermal Trajectory, **e)** $ROTC_{SS}$, and **f)** $DTR_{30}$.

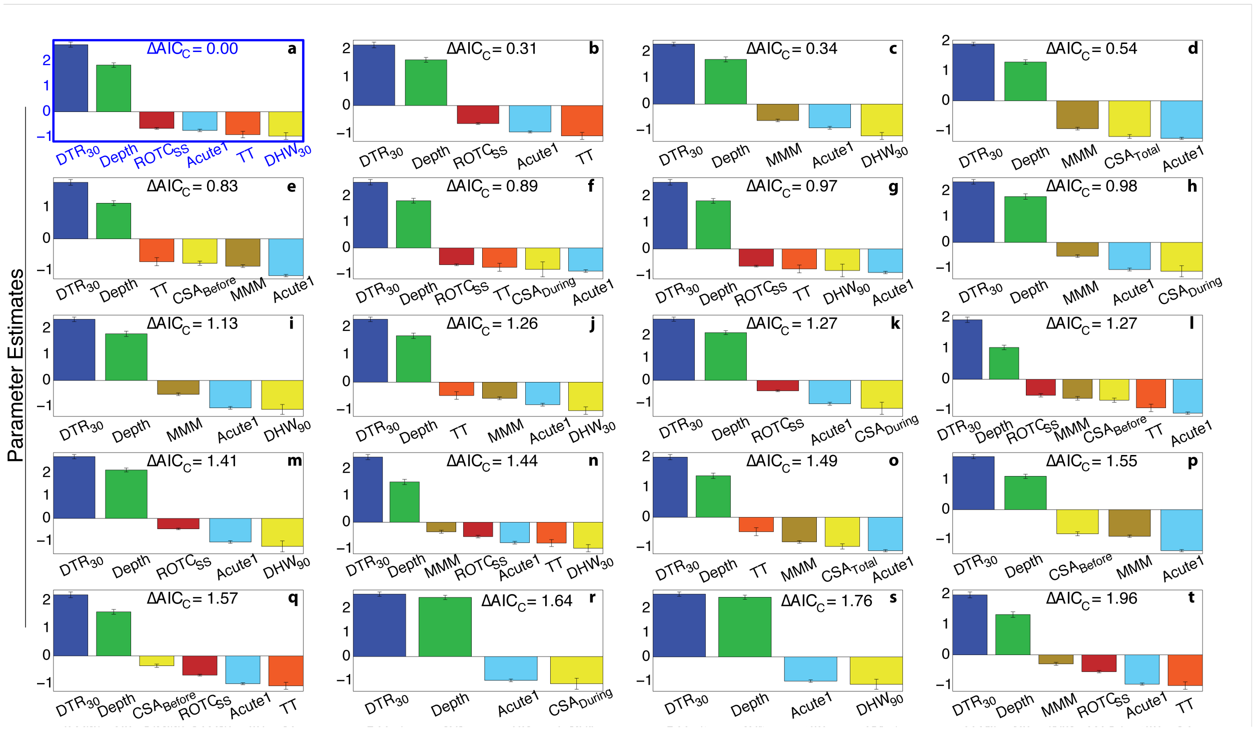

**Supplementary Figure 6| All *in situ*-based logit models within $\Delta AIC_C \leq 2$ of the highest ranked model.** Standardized model coefficients (y-axis) and their corresponding explanatory variable (x-axis), with jackknifed standard error bars and $\Delta AIC_C$ values indicated. **(a)** The global best model with $\Delta AIC_C = 0$; **(b-t)** all models within $\Delta AIC_C \leq 2$, which are statistically indistinguishable from **(a)**.

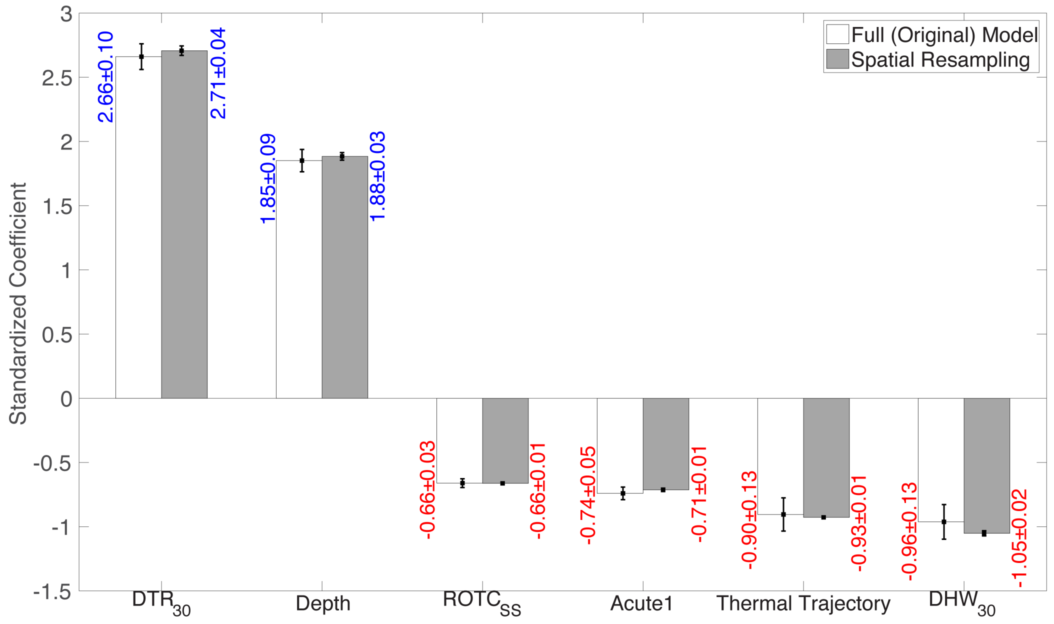


**Supplementary Figure 7| Highest-ranked *in situ* model results after spatial resampling.** Standardized coefficients for the highest-ranked model parameter estimates fit to the entire data set are shown in white (± s.e.), while the average of the resampled parameter estimates for the same covariates are shown in grey (± s.e.). The modified jackknife resampling scheme involved grouping sites within 10 km of each other and removing each of these proximity groups incrementally before fitting OLR models to the remaining data.

| Interaction Term | Interaction Coefficient | Coefficient for: $DHW_{30}$ | $DTR_{30}$ | $AIC_C$ | $\Delta AIC_C$ |
|---|---|---|---|---|---|
| Depth x $DHW_{30}$ | 1.78 | 2.11 | -2.31 | 143.57 | -0.18 |
| Depth x $DTR_{30}$ | 2.69 | 1.77 | -1.72 | 142.74 | -1.01 |
| Depth x $ROTC_{SS}$ | -0.09 | 0.95 | -2.63 | 146.32 | 2.57 |
| Depth x MMM | -0.11 | 0.96 | -2.6 | 146.31 | 2.56 |
| MMM x $DHW_{30}$ | 0.8 | 2.04 | -2.85 | 144.41 | 0.66 |
| MMM x TT | -0.4 | 0.83 | -2.89 | 145 | 1.25 |
| MMM x Acute1 | 0.32 | 1.14 | -2.8 | 145.3 | 1.55 |
| MMM x $ROTC_{SS}$ | -0.01 | 0.97 | -2.67 | 146.35 | 2.60 |
| Acute1 x $DHW_{30}$ | -3.94 | 5.99 | -3.06 | 143.37 | -0.38 |
| TT x $DHW_{30}$ | -0.55 | 2.1 | -2.51 | 144.27 | 0.52 |
| $ROTC_{SS}$ x $DTR_{30}$ | -0.15 | 0.97 | -2.63 | 146.32 | 2.57 |

**Supplementary Table 1: Nonlinear Ordinal Logistic Regression Models.** *In situ* variable interaction terms added to the highest-ranked OLR model (Fig. 3b), coefficient parameter estimates for these interaction terms, the corresponding $DHW_{30}$ and $DTR_{30}$ coefficient parameter estimates, and $AIC_C$ and $\Delta AIC_C$ values of the resulting models.

## Supplementary References


1. McClanahan, T. R. & Maina, J. Response of Coral Assemblages to the Interaction between Natural Temperature Variation and Rare Warm-Water Events. Ecosystems 6, 551-563, doi:10.1007/s10021-002-0104-x (2003).

2. McClanahan, T. R. et al. Predictability of coral bleaching from synoptic satellite and in situ temperature observations. Coral Reefs 26, 695-701, doi:10.1007/s00338-006-0193-7 (2007).

3. Conand, F., Marsac, F., Tessier, E. & Conand, C. A ten-year period of daily sea surface temperature at a coastal station in Reunion Island, Indian Ocean (July 1993–April 2004): patterns of variability and biological responses. Western Indian Ocean Journal of Marine Science 6 (2008).

4. Oliver, T. & Palumbi, S. Do fluctuating temperature environments elevate coral thermal tolerance? Coral Reefs 30, 429-440 (2011).

5. Oliver, T. A. & Palumbi, S. R. Distributions of stress-resistant coral symbionts match environmental patterns at local but not regional scales. Marine Ecology Progress Series 378, 93-103, doi:10.3354/meps07871 (2009).

6. Williams, G. J., Knapp, I. S., Maragos, J. E. & Davy, S. K. Modeling patterns of coral bleaching at a remote Central Pacific atoll. Mar Pollut Bull 60, 1467-1476, doi:10.1016/j.marpolbul.2010.05.009 (2010).

7. Rogers, J. S., Monismith, S. G., Koweek, D. A., Torres, W. I. & Dunbar, R. B. Thermodynamics and hydrodynamics in an atoll reef system and their influence on coral cover. Limnology and Oceanography 61, 2191-2206, doi:10.1002/lno.10365 (2016).

8. Davis, K. A. & Monismith, S. G. The Modification of Bottom Boundary Layer Turbulence and Mixing by Internal Waves Shoaling on a Barrier Reef. Journal of Physical Oceanography 41, 2223-2241, doi:10.1175/2011jpo4344.1 (2011).

9. Manzello, D. P., Berkelmans, R. & Hendee, J. C. Coral bleaching indices and thresholds for the Florida Reef Tract, Bahamas, and St. Croix, US Virgin Islands. Mar Pollut Bull 54, 1923-1931, doi:10.1016/j.marpolbul.2007.08.009 (2007).

10. Edmunds, P. J. Decadal-scale changes in the community structure of coral reefs of St. John, US Virgin Islands. Marine Ecology Progress Series 489, 107-123, doi:10.3354/meps10424 (2013).

11. Australian Institute of Marine Science (AIMS). 2017, Sea Water Temperature Logger Data at Bird Island, From 14 Feb 2013 To 04 Feb 2016, http://data.aims.gov.au/metadataviewer/faces/view.xhtml?uuid=bf04b7d7-583e-4cff-bf1f-e5ed5add34c1, accessed 27/04/2017

12. Australian Institute of Marine Science (AIMS). 2017, Sea Water Temperature Logger Data at Border Island, From 24 Mar 2011 To 18 Jan 2015, http://data.aims.gov.au/metadataviewer/faces/view.xhtml?uuid=c3a08a7e-c29d-4a38-9e61-c2cd1a996b49, accessed 27/04/2017

13. Australian Institute of Marine Science (AIMS). 2017, Sea Water Temperature Logger Data at Cattle Bay, Great Barrier Reef From 11 Feb 1993 To 06 Jun 2016, http://data.aims.gov.au/metadataviewer/faces/view.xhtml?uuid=c6d2c182-7c07-4d7b-b9d4-a5b9c73f8aaa, accessed 27/04/2017

14. Australian Institute of Marine Science (AIMS). 2017, Sea Water Temperature Logger Data at Chicken Reef, Great Barrier Reef From 27 Apr 1996 To 30 Jul 2016, http://data.aims.gov.au/metadataviewer/faces/view.xhtml?uuid=f0436176-ee83-4f3a-babd-34eadd6d140d, accessed 27/04/2017

15. Australian Institute of Marine Science (AIMS). 2017, Sea Water Temperature Logger Data at Davies Reef, Great Barrier Reef From 02 May 1996 To 28 Mar 2016, http://data.aims.gov.au/metadataviewer/faces/view.xhtml?uuid=0ba80e4d-eeb9-4b34-9fff-fc6d2787bad1, accessed 27/04/2017

16. Australian Institute of Marine Science (AIMS). 2017, Sea Water Temperature Logger Data at Daydream Island, Great Barrier Reef From 26 Jun 1996 To 06 May 2016, http://data.aims.gov.au/metadataviewer/faces/view.xhtml?uuid=9ea12078-6571-4baf-9d47-e91c556967c2, accessed 27/04/2017

17. Australian Institute of Marine Science (AIMS). 2017, Sea Water Temperature Logger Data at Dent Island, Great Barrier Reef From 18 Dec 2005 To 11 Jul 2015, http://data.aims.gov.au/metadataviewer/faces/view.xhtml?uuid=f2a2d747-06cf-4d24-b9cd-310ca719216e, accessed 27/04/2017

18. Australian Institute of Marine Science (AIMS). 2017, Sea Water Temperature Logger Data at Geoffrey Bay, Great Barrier Reef From 20 Nov 1991 To 08 Jun 2016, http://data.aims.gov.au/metadataviewer/faces/view.xhtml?uuid=cb760466-76dc-4e32-84b6-a7bd5a8ef806, accessed 27/04/2017

19. Australian Institute of Marine Science (AIMS). 2017, Sea Water Temperature Logger Data at Halfway Island, Great Barrier Reef From 07 Dec 1995 To 11 Aug 2013, http://data.aims.gov.au/metadataviewer/faces/view.xhtml?uuid=9a661fb5-ba2f-4b7f-8e9d-d3d007932eff, accessed 27/04/2017

20. Australian Institute of Marine Science (AIMS). 2017, Sea Water Temperature Logger Data at Hardy Reef, Great Barrier Reef From 13 Jun 2002 To 20 Nov 2002, http://data.aims.gov.au/metadataviewer/faces/view.xhtml?uuid=fc73ac05-a4a7-4bcb-931d-c246f47aed93, accessed 27/04/2017

21. Australian Institute of Marine Science (AIMS). 2017, Sea Water Temperature Logger Data at Heron Island, Great Barrier Reef From 24 Nov 1995 To 30 Mar 2015, http://data.aims.gov.au/metadataviewer/faces/view.xhtml?uuid=446a0e73-7c30-4712-9ddb-ba1fc29b8b9a, accessed 27/04/2017

22. Australian Institute of Marine Science (AIMS). 2017, Sea Water Temperature Logger Data at Kelso Reef, Great Barrier Reef From 04 Jul 1995 To 02 Jul 2015, http://data.aims.gov.au/metadataviewer/faces/view.xhtml?uuid=43950f6a-0ee7-4291-bed8-c6f9a90ce15f, accessed 27/04/2017

23. Australian Institute of Marine Science (AIMS). 2017, Sea Water Temperature Logger Data at Low Isles, Great Barrier Reef From 30 Mar 1996 To 05 Sep 2016, http://data.aims.gov.au/metadataviewer/faces/view.xhtml?uuid=ef62b2ad-bfd2-41b3-8c1d-c89715052c64, accessed 27/04/2017

24. Australian Institute of Marine Science (AIMS). 2017, Sea Water Temperature Logger Data at Miall, Great Barrier Reef From 27 Apr 2010 To 02 Mar 2011, http://data.aims.gov.au/metadataviewer/faces/view.xhtml?uuid=c7ffcbab-813f-469f-be0c-25a434f78612, accessed 27/04/2017

25. Australian Institute of Marine Science (AIMS). 2017, Sea Water Temperature Logger Data at Middle Reef, Great Barrier Reef From 25 Jan 1996 To 02 Nov 2012, http://data.aims.gov.au/metadataviewer/faces/view.xhtml?uuid=42994f7d-ddef-4509-9fef-49d6fc8e246b, accessed 27/04/2017

26. Australian Institute of Marine Science (AIMS). 2017, Sea Water Temperature Logger Data at Middle Island, Great Barrier Reef From 07 Dec 1995 To 15 Jul 1997, http://data.aims.gov.au/metadataviewer/faces/view.xhtml?uuid=b7195bb8-d017-4d8a-9778-e164b6eae835, accessed 27/04/2017

27. Australian Institute of Marine Science (AIMS). 2017, Sea Water Temperature Logger Data at Myrmidon Reef, Great Barrier Reef From 13 Apr 1995 To 04 Jun 2015, http://data.aims.gov.au/metadataviewer/faces/view.xhtml?uuid=1342bfa4-57e9-4d8a-ac77-1ec34ec2d16b, accessed 27/04/2017

28. Australian Institute of Marine Science (AIMS). 2017, Sea Water Temperature Logger Data at Nelly Bay, Great Barrier Reef From 25 Jan 1996 To 02 Nov 2012, http://data.aims.gov.au/metadataviewer/faces/view.xhtml?uuid=0e781a14-fbac-482d-afdd-e348c4996ea9, accessed 27/04/2017

29. Australian Institute of Marine Science (AIMS). 2017, Sea Water Temperature Logger Data at Norman Reef, Great Barrier Reef From 25 Mar 1996 To 11 Aug 2011, http://data.aims.gov.au/metadataviewer/faces/view.xhtml?uuid=56dd3569-eb17-466c-b139-7ea491d5b4b7, accessed 27/04/2017

30. Australian Institute of Marine Science (AIMS). 2017, Sea Water Temperature Logger Data at North Keppel Island, Great Barrier Reef From 07 Dec 1995 To 13 May 2015, http://data.aims.gov.au/metadataviewer/faces/view.xhtml?uuid=d825864f-e3a1-4465-9efb-aeb581458610, accessed 27/04/2017

31. Australian Institute of Marine Science (AIMS). 2017, Sea Water Temperature Logger Data at Orpheus Island, Great Barrier Reef From 07 Jul 2011 To 16 Apr 2015, http://data.aims.gov.au/metadataviewer/faces/view.xhtml?uuid=b3035761-4ea4-4bdd-a64e-45871a9c94bb, accessed 27/04/2017

32. Australian Institute of Marine Science (AIMS). 2017, Sea Water Temperature Logger Data at Pelorus Island, Great Barrier Reef From 04 Aug 1993 To 19 Sep 2016, http://data.aims.gov.au/metadataviewer/faces/view.xhtml?uuid=6f58a3d8-6c3d-4e22-8a9e-4cdf08e861ca, accessed 27/04/2017

33. Australian Institute of Marine Science (AIMS). 2017, Sea Water Temperature Logger Data at Pioneer Bay, Great Barrier Reef From 08 Mar 1992 To 07 Jul 2011, http://data.aims.gov.au/metadataviewer/faces/view.xhtml?uuid=594050fd-fe1e-4921-9b87-4ccd4835e974, accessed 27/04/2017

34. Australian Institute of Marine Science (AIMS). 2017, Sea Water Temperature Logger Data at Wallace Islet, Great Barrier Reef From 21 Nov 1996 To 23 Oct 2013, http://data.aims.gov.au/metadataviewer/faces/view.xhtml?uuid=dc97de8f-a9e9-4922-99c2-30b29e376f46, accessed 27/04/2017

35. Davis, K. A. et al. Observations of the thermal environment on Red Sea platform reefs: a heat budget analysis. Coral Reefs 30, 25-36, doi:10.1007/s00338-011-0740-8 (2011).

36. Pineda, J. et al. Two spatial scales in a bleaching event: Corals from the mildest and the most extreme thermal environments escape mortality. Limnology and Oceanography 58, 1531-1545, doi:10.4319/lo.2013.58.5.1531 (2013).

37. McClanahan, T., Muthiga, N. & Mangi, S. Coral and algal changes after the 1998 coral bleaching: interaction with reef management and herbivores on Kenyan reefs. *Coral Reefs* **19**, 380-391, doi:10.1007/s003380000133 (2014).

38. McClanahan, T. R., Ateweberhan, M., Muhando, C. A., Maina, J. & Mohammed, M. S. Effects of climate and seawater temperature variation on coral bleaching and mortality. *Ecological Monographs* **77**, 503-525 (2007).

39. Berkelmans, R. & Oliver, J. Large-scale bleaching of corals on the Great Barrier Reef. *Coral reefs* **18**, 55-60 (1999).

40. Berkelmans, R. Time-integrated thermal bleaching thresholds of reefs and their variation on the Great Barrier Reef. *Marine ecology progress series* **229**, 73-82 (2002).

41. Berkelmans, R., De'ath, G., Kininmonth, S. & Skirving, W. J. A comparison of the 1998 and 2002 coral bleaching events on the Great Barrier Reef: spatial correlation, patterns, and predictions. *Coral reefs* **23**, 74-83 (2004).

42. Berkelmans, R., Jones, A. & Schaffelke, B. Salinity thresholds of Acropora spp. on the Great Barrier Reef. *Coral Reefs* **31**, 1103-1110 (2012).

43. D. Elvidge, C. *et al.* Satellite observation of Keppel Islands (Great Barrier Reef) 2002 coral bleaching using IKONOS data. *Coral Reefs* **23**, 123-132, doi:10.1007/s00338-003-0364-8 (2004).

44. Maynard, J. A. *et al.* ReefTemp: An interactive monitoring system for coral bleaching using high-resolution SST and improved stress predictors. *Geophysical Research Letters* **35**, doi:10.1029/2007gl032175 (2008).

45. Maynard, J. A., Anthony, K. R. N., Marshall, P. A. & Masiri, I. Major bleaching events can lead to increased thermal tolerance in corals. *Marine Biology* **155**, 173-182, doi:10.1007/s00227-008-1015-y (2008).

46. Marshall, P. & Baird, A. Bleaching of corals on the Great Barrier Reef: differential susceptibilities among taxa. *Coral reefs* **19**, 155-163 (2000).

47. Furby, K. A., Bouwmeester, J. & Berumen, M. L. Susceptibility of central Red Sea corals during a major bleaching event. *Coral Reefs* **32**, 505-513 (2013).
