# Supplementary material for: High frequency temperature variability reduces the risk of coral bleaching
Source: Nat Commun. 2018 Apr 26;9:1671. doi: 10.1038/s41467-018-04074-2 (PMC5920114; doi:10.1038/s41467-018-04074-2)
Supplement: Supplementary file 3 — Description of Additional Supplementary Files [file 41467_2018_4074_MOESM3_ESM.pdf]

Descriptions of Additional Supplementary Files

File Name: Supplementary Data 1

Description: A table detailing the metadata of all of the temperature time series data we use in our study. This includes locations, coordinates, logger depths and date ranges for the many different temperature recording instruments.

File Name: Supplementary Data 2

Description: An Excel file detailing the locations, dates, and prevalence scores (as defined in the Methods section of the Main Text of our manuscript) of 81 distinct observations of coral bleaching. As many of these observations are derived from prior publications, references to those respective publications are also included.

File Name: Supplementary Data 3

Description: An Excel file created in response to a comment made by a Reviewer, in which the Reviewer compelled us to repeat our ordinal logistic regression analysis with the inclusion of a random effect accounting for reef proximities to each other. This table details the results of the ensuing mixed effects ordinal logistic regression analysis, including parameter estimates for the covariates incorporated in each model, parameter estimates for the random effect term in each model, as well as metrics for model selection and assessing the predictive power of each model.
